# Associations Between Multiple Leisure Activities, Mental Health and Substance Use Among Adolescents in Denmark: A Nationwide Cross-Sectional Study

**DOI:** 10.3389/fnbeh.2020.593340

**Published:** 2020-12-21

**Authors:** Ziggi Ivan Santini, Charlotte Meilstrup, Carsten Hinrichsen, Line Nielsen, Ai Koyanagi, Vibeke Koushede, Ola Ekholm, Katrine Rich Madsen

**Affiliations:** ^1^The National Institute of Public Health, University of Southern Denmark, Copenhagen, Denmark; ^2^Parc Sanitari Sant Joan de Déu, Fundació Sant Joan de Déu, Centro de Investigación Biomédica en Red de Salud Mental (CIBERSAM), Universitat de Barcelona, Barcelona, Spain; ^3^Catalan Institution for Research and Advanced Studies (ICREA), Barcelona, Spain; ^4^Department of Psychology, University of Copenhagen, Copenhagen, Denmark

**Keywords:** adolescence, leisure activity, substance use, mental health, public health

## Abstract

**Background:** Previous research has suggested that leisure activity may benefit mental health and protect against substance use among adolescents, but more research is needed to asses associations with a wide range of outcomes. The aim of this study was to assess associations between multiple leisure activities and (1) mental health outcomes and (2) substance use outcomes in a sample of Danish adolescents.

**Methods:** Using data from the Danish part of the European School Survey Project on Alcohol and Other Drugs (ESPAD) collected in 2019, nation-wide cross-sectional data from 2,488 participants aged 15 or 16 in Denmark were analyzed to assess associations between number of leisure activity types and outcomes pertaining to mental health and substance use.

**Results:** Our results show that engaging in multiple activity types at least once a week—as compared to one single type of activity—is associated with increased odds for high mental well-being, and reduced odds for mental health problems. Engaging in multiple activity types is also associated with reduced odds for overall substance use and for using substances as a coping method. Among those using substances, engaging in multiple activity types is associated with reduced odds of above average substance use.

**Conclusion:** Increasing opportunities for adolescents to engage in leisure activities is suggested to be useful in enhancing mental health and preventing substance use and promoting mental health. Promoting and increasing access to leisure activities among adolescents could be a promising avenue for policy and practice.

## Introduction

In recent years, Denmark has seen an increase in the proportion of young adults and adolescents with poor mental health (Due et al., 2014; Jensen et al., 2018). Further, substance use, such as alcohol consumption and smoking, continues to be a pervasive problem among adolescents in Denmark (Pisinger et al., 2019; WHO, 2020). For example, 82% of Danish 15-year-olds have consumed alcohol, whereas the European average for the same age group is 59% (WHO, 2020). Further, 65% of Danish 15-year-olds have consumed alcohol within the last 30 days (compared to a European average of 37%), and 42% have been intoxicated at least twice (compared to 20% among the European counterpart) (WHO, 2020). In regards to tobacco consumption and cannabis use within the past 30 days, 15% of European adolescents report having smoked cigarettes, while 7% report having used cannabis (WHO, 2020). Adolescent substance use is of major concern because of the health-risk behaviors associated with substance use (e.g., dangerous behaviors, violence, unsafe sex) (DuRant et al., 1999; Hjarnaa et al., 2020), the adverse health consequences (including elevated risks of mortality and suicidal behaviors) both occurring in adolescence and later in life (Bonnie et al., 2004; Esposito-Smythers and Spirito, 2004; Clark et al., 2008), the strong co-occurrence with mental health problems (Storr et al., 2012; Schulte and Hser, 2013), and because adolescent substance use has prognostic significance in the development of mental health problems and substance use disorders throughout adolescence and into adulthood (Brook et al., 2002; Stone et al., 2012; Wymbs et al., 2014). Similarly, mental health problems occurring in adolescence is predictive of mental and substance use disorders in adulthood (Copeland et al., 2009; Ning et al., 2020). It is therefore critical to explore factors that protect and promote good mental health and healthy lifestyles in adolescence.

Engagement in leisure activities is generally considered an indicator of relational health and social well-being, since such activities often promote or reinforce shared interests among adolescents and their peers, as well as social interaction during these activities (Zeijl et al., 2000; Caldwell and Faulk, 2013). Research suggests that engaging in leisure activities—particularly those that require effort, concentration, or commitment—is essential in the protection and promotion of good mental health (Trainor et al., 2010; Santini et al., 2017a, 2018a). This is also supported in research on adolescents as well as school-based interventions (Bartko and Eccles, 2003; Palen and Coatsworth, 2007; Trainor et al., 2010; Anwar McHenry et al., 2018). Leisure activities are thought to be developmentally important because they can provide new skills and foster self-esteem, a sense of meaning, and social connectedness during adolescence, all of which are conducive to mental well-being and resilience (Kleiber and Kirshnit, 2002; Santini et al., 2020). Leisure activities may also serve as a buffer against stress, which implies that such activities can be a method for coping with challenges in a healthy way (Kleiber, 1999; Iwasaki and Mannell, 2000; Trenberth and Dewe, 2002). This is relevant in terms of mental health and substance use, since human beings are pain and stress aversive, and may—in lack of healthy alternatives—resort to substance use as a maladaptive way to cope with stressors and difficult emotional states (Wills and Filer, 1996).

Behavioral economic theories have been applied in research on alcohol and substance use and misuse and have made contributions to characterizing how the presence of alternative reinforcers affect the consumption of such substances (Vuchinich et al., 1987; Vuchinich and Tucker, 1988). According to this theoretical approach, individuals are less likely to consume a substance if they are confronted with a number of alternative reinforcers (i.e., rewarding alternatives to substance use, which makes it likely that the person will repeat this behavior), particularly if such reinforcers afford greater long-term advantages. Another relevant theory pertains to that of an “opportunity cost,” which refers to a situation where options are mutually exclusive, i.e., the selection of one option renders the other option unavailable. When applied to alcohol and substance use, the consumption of substances requires time and resources, and individuals are less likely to use or misuse alcohol or other substances if their time and resources are focused elsewhere (e.g., sports, hobbies, social interaction, etc.) (Bickel et al., 2014). In terms of adolescents and young adults specifically, studies have shown that activity engagement can effectively protect against the development of alcohol and drug misuse (Murphy et al., 2006; Audrain-McGovern et al., 2013; Leventhal et al., 2015).

Much of the evidence consist mainly of isolated types of activities (mostly physical activity), while other types of leisure activities are not included. Further, similar research studies conducted on adolescent samples in Scandinavia are scarce, which means that studies are needed to replicate previous findings in the Scandinavian setting, particularly comprehensive studies that cover a wide range of outcomes pertaining to mental health and substance use. Thus, the aim of the current study is to assess associations between multiple leisure activities and (1) mental health outcomes and (2) substance use outcomes on a large sample of Danish adolescents. Based on the literature reviewed, we hypothesized that individuals that engage in multiple activity types would show a reduced likelihood of poor mental health and engagement in substance use, as compared to individuals that engage in just one type of activity. We further hypothesized that among those using substances, individuals engaging in multiple activity types would show a reduced likelihood of above average substance use, as well as reduced likelihood of using substances as a means of coping.

## Methods

### Study Design

Data stem from the Danish contribution to the European School Survey Project on Alcohol and Other Drugs (ESPAD) in 2019, which is a cross-sectional survey among adolescent students conducted in numerous European countries. The survey is conducted every 4 years. The ESPAD target population is defined as students who turn 16 in the calendar year of the survey (i.e., students may be 15 or 16 years old when filling out the questionnaire) and are present in the classroom on the day the survey was conducted. Statistical data and comprehensive methodological documentation are freely available on the website of the ESPAD (www.espad.org). A cluster sampling design was used to sample the target population. The target population for ESPAD included students who were enrolled in regular, vocational, general or academic studies were included. It did not include those who were enrolled in either special schools or special classes for students with learning disorders or severe physical disabilities. The school participation rate was 21% (a total of 81; 158 classes). Within the participating schools, the proportion of students that were present on the day of the data collection was 87% and all present students completed the questionnaire. Data were collected by a self-administered electronic questionnaire. The students answered the questionnaires anonymously in the classroom, with teachers functioning as survey leaders. The sample size for the Danish data was 2,488 students.

### Measures

#### Outcomes: Mental Health and Substance Use

For the analyses, we used a numerous outcomes outcomes pertaining to mental health and substance use. All outcomes are briefly mentioned here and listed in Table 1. All outcomes are further described in detail in Appendix 1. Mental health outcomes included mental health problems (very bothered by any of four symptoms vs. else) as well as high mental well-being (measured using SWEMWBS—cut-point top 15% vs. below).

**Table 1:** List of outcomes.

**Outcome**	**Assessed among the total sample**	**Assessed among a subsample**
Mental health	Mental health problems (very bothered)	
	High mental well-being	
Smoking	Any cigarette smoking	Above average cigarette smoking
	Any consumption of other forms of tobacco	
Alcohol consumption	Any alcohol consumption	Above average alcohol consumption
	Any binge drinking	Above average binge drinking
	Any occasions being intoxicated	Above average occasions of being intoxicated
	Any coping with alcohol	
Drug and cannabis use	Any drug use	
	Any cannabis consumption	Above average cannabis consumption

Smoking outcomes included any current cigarette smoking (yes vs. no), and having smoked e-cigarettes, waterpipe, or heat-not-burn tobacco within the past 12 months (any consumption vs. none). Among those who smoked cigarettes, we created a dichotomous variable on the number of cigarettes smoked per day—more than average or not (above average was 1 cigarette per day or more).

General alcohol consumption referred to having consumed any alcohol within the past 30 days (yes vs. no), and among those who did, a variable on being in the group of above average drinking occasions within the past 30 days was created (above average was 6 occasions or more). The following alcohol consumption variables applied only to those who had consumed alcohol within the past 30 days. Binge drinking referred to having consumed five or more drinks on one occasion within the past 30 days (yes or no), and among those who had engaged in binge drinking, a variable on being in the group of above average binge drinking occasions was created (above average was 3 occasions or more). Intoxication referred to any occasions of having been intoxicated from drinking alcoholic beverages within the past 30 days (yes vs. no), while we also created another variable on whether the participant reported below or above average occasions of being intoxicated (above average was 3 occasions or more). Consuming alcohol as a coping method within the past 12 months was operationalized as reporting using alcohol to forget problems, to cheer up, or as an antidepressant (minimum responding “seldom” to at least one of the three items vs. less than seldom on all three or none at all).

Drug consumption was defined as ever having used ecstasy, amphetamines, methamphetamines, cocaine, crack, heroin, inhalants, LSD, or some other hallucinogens, magic mushrooms, GHB, drugs by injection with a needle, tranquilizers, or sedatives, alcohol together will pills (medicaments) in order to get high, painkillers in order to get high, or nitrous oxide cartridges (consumption of at least one of these drugs vs. never).

Cannabis consumption included having used any cannabis within the past 12 months (yes vs. no), and among those who did, a variable on being in the group of above average cannabis consumption within the past 12 months was created (above average was 6 occasions or more).

#### Predictor: Number of Leisure Activity Types

Participants were asked how often they engaged in four different leisure activities: (1) playing computer games, (2) actively participating in sports, athletics, or exercising; (3) reading books for enjoyment (not counting school books), and (4) other hobbies (playing an instrument, singing, drawing, writing, etc.). Possible response categories were as follows: never; a few times a year; once or twice a month; at least once a week; almost every day. Since we were interested in the incremental effect of engaging in a variety of leisure activities, we generated a combined categorical scale to reflect the number of leisure activities (out of the four) that participants engaged in. Further, since the vast majority of participants engaged in activities at least weekly, the scale was constructed with the main categories reflecting this, and one minority category reflecting less than weekly activity. Thus, our constructed scale for “number of leisure activity types” was operationalized with the following coding: 0 = active less than weekly in any number of activities or not at all; 1 = active in one activity at least once a week (used as a reference category); 2 = active in two activities at least once a week; 3 = active in three or four activities at least once a week. The resulting four categories and the number of participants in each can be observed in Table 2. The second category “active in one activity at least once a week” was chosen as a reference category based on our hypothesis that engagement in multiple activity types (vs. one type) would play a role in relation to the outcomes, and also because it was the first category after the non-normative category 0 (i.e., non-normative in the sense of being an absolute minority relative to the other categories). While there is no consensus in terms of validating combined items for leisure participation, it is common to construct variables based on summary indices or the coding of variables based on weekly participation (Verghese et al., 2003; Leversen et al., 2012; Santini et al., 2017a,b, 2018b).

**Table 2:** Characteristics of the study sample.

**Characteristic**	**Category**	***N***	**Weighted %**
Total participants		2,488	
Sex	Female	1,303	52.5
Father's education	Completed primary school or less	40	1.6
	Some secondary school	272	10.8
	Completed secondary school	499	19.9
	Some college or university	463	18.5
	Completed college or university	671	27.5
	Missing	543	21.8
Mother's education	Completed primary school or less	14	0.6
	Some secondary school	118	0.5
	Completed secondary school	514	20.4
	Some college or university	768	30.7
	Completed college or university	662	27.1
	Missing	412	16.4
Family support	Continuous scale (range 1–7), mean (SD)	5.9 (1.7)	
Availability of friends	Continuous scale (range 1–7), mean (SD)	5.7 (1.7)	
Parental relative income status	Better off	1,609	66.5
	About the same	658	26.9
	Less well-off	160	6.7
Number of leisure activity types	Active less than weekly or not at all	91	3.7
	Active in one activity at least once a week	695	28.3
	Active in two activities at least once a week	992	40.5
	Active in three/four activities at least once a week	666	27.5
Symptoms of mental health problems and discomfort	Continuous scale (range 0–10), mean (SD)	3.7 (2.6)	
	Very bothered by any symptom—present	1,006	40.8
Mental well-being	Continuous scale (range 7–35), mean (SD)	22.7 (4.7)	
	High mental well-being—present	323	14.1
Any cigarette smoking	Present	547	22.5
Any consumption of other forms of tobacco	Present	689	27.5
Any cannabis consumption	Present	364	15.1
Any alcohol consumption	Present	1,823	74.1
Any binge drinking^a^	Present	1,437	79.0
Any occasions being intoxicated^a^	Present	972	54.3
Any coping with alcohol^b^	Present	1,088	50.2
Any drug use	Present	341	14.0

*Data in column N are the number of individuals in the sample unless otherwise specified (when mean and standard deviations are reported instead). Sampling weights were used for the calculation of proportions and means (SD)*.

a *The sample was restricted to respondents that reported consuming alcohol on any number of occasions other than 0 within the past 30 days (i.e., sample n = 1,823)*.

b *The sample was restricted to respondents that reported consuming alcohol on any number of occasions other than 0 within the past 12 months (i.e., sample n = 2,180)*.

#### Covariates

Demographic characteristics included sex but not age, since all participants were born in 2003. Parental level of education was assessed by asking respondents about the mother's and father's education. Possible categories were: (1) completed primary school or less; (2) some secondary school; (3) completed secondary school, (4) some college, or university; (5) completed college or university. If both mother's and father's education were reported, the mean was used. If only one was reported, only the reported one was used. Because 23.4% of the data on parental education were missing, missing categories were created for mother's and father's education (shown in Table 2). Parental relative income status was assessed by asking the participant “How well-off is your family compared to other families in your country?,” with the following categorization of responses: (1) better off; (2) about the same; (3) less well-off. To assess family support, we used the following item: “I get the emotional help and support I need from my family.” Availability of friends was assessed with the item “I have friends with whom I can share my joys and sorrows.” Responses for family support and availability of friends ranged from 1 “very strongly disagree” to 7 “very strongly agree.” Finally, a continuous measure for symptoms of mental health problems (see Appendix 1) was also added as a covariate in most models (described below).

#### Statistical Analysis

The statistical analysis was done with Stata version 13.1 (Stata Corp LP, College Station, Texas). A descriptive analysis was conducted to demonstrate characteristics of the population. These analyses included unweighted frequencies, and weighted proportions, means, and standard deviations. Multivariable logistic regression analyses were conducted to assess the associations between number of leisure activity types and all outcomes. In the model with mental health problems (binary) as the outcome, covariates were gender (binary); parental education (categorical); family support (continuous), availability of friends (continuous), parental financial status (categorical). All other models adjusted for the aforementioned covariates as well as symptoms of mental health problems (continuous, see Appendix 1). The latter was added as a covariate since mental health problems may influence leisure activity involvement (Duncan et al., 2018) as well as mental well-being and substance use (Wills and Filer, 1996). The statistical models conducted pertained to two types of estimations: (1) models conducted to estimate the odds of an outcome across the entire sample (e.g., odds of smoking overall), and (2) models conducted to estimate the odds of an outcome among a restricted sub-sample (e.g., odds of above average smoking among those who smoke). All statistical models were based on the sample with no missing data (complete case analysis). Information regarding the proportion of missing data can be found in Appendix 1. In order to adjust the sample to the sociodemographic composition of the target population, the complex data design was taken into account in all analyses using the svy function, including clustering within schools and classes. Results are expressed as coefficients or odds ratio coefficients (OR) and 95% confidence intervals (95% CIs). A *p* < 0.05 was considered to be statistically significant.

## Results

Table 2 shows the characteristics of the study sample. In terms of leisure activities, 1,043 (45.9%) played computer games at least weekly, 2,174 (87.5%) engaged in sports, athletics or exercising at least weekly, 474 (19.5%) read books for enjoyment at least weekly, and 1,035 (42.3%) engaged in other hobbies at least weekly. A minority of participants (3.7%) engaged less than weekly in any number of activities or not at all, while 28.3% engaged in one activity at least weekly, 40.5% engaged in two activities at least weekly, and 27.5% engaged in three or four activities at least weekly. Below, we report only the analytical results that were statistically significant (*p* < 0.05).

Table 3 displays associations between number of leisure activity types and mental health outcomes. Less than weekly activity was not associated with any of the mental health outcomes, as compared to being active in one activity at least weekly. Being active in three or four activities at least weekly was associated with 27% reduced odds of being very bothered by mental health problems, as well as 62% increased odds of high mental well-being, both as compared to being active in one activity at least weekly.

**Table 3:** The association between number of leisure activity types and mental health estimated by multivariable logistic regression.

	**OR**	**95% CI**	***P*-value**
	Mental health problems (very bothered)^a^		
Number of leisure activity types			
- Active less than weekly or not at all	1.20	0.80, 1.82	0.376
- Active in one activity at least once a week	1		
- Active in two activities at least once a week	0.83	0.68, 1.03	0.089
- Active in three or four activities at least once a week	0.73	0.57, 0.95	0.020
	High mental well-being^b^		
Number of leisure activity types			
- Active less than weekly or not at all	0.66	0.23, 1.95	0.451
- Active in one activity at least once a week	1		
- Active in two activities at least once a week	1.10	0.78, 1.56	0.594
- Active in three or four activities at least once a week	1.62	1.19, 2.22	0.003

*OR, odds ratio; CI, confidence interval*.

a *The model adjusted for gender, parental education, and parental financial status, family support, and availability of friends*.

b *The model adjusted for gender, parental education, and parental financial status, family support, availability of friends, and symptoms of mental health problems*.

Table 4 displays associations between the number of leisure activity types and smoking (cigarette and other forms of tobacco). Less than weekly activity was associated with more than double the odds of smoking as well as above average smoking among participants that smoked. Engaging in two or more activities at least weekly was associated with 38–57% reduced odds of smoking cigarettes, as well as 53–60% reduced odds of smoking more cigarettes than average among those who smoked cigarettes. Engaging in two or more activities was also associated with 47–62% reduced odds of alternative forms of tobacco (e-cigarettes, waterpipe, heat-not-burn tobacco).

**Table 4:** The association between number of leisure activity types and smoking estimated by multivariable logistic regression.

	**OR**	**95% CI**	***P*-value**
	Any cigarette smoking		
Number of leisure activity types			
- Active less than weekly or not at all	2.16	1.30, 3.60	0.004
- Active in one activity at least once a week	1		
- Active in two activities at least once a week	0.62	0.46, 0.84	0.003
- Active in three or four activities at least once a week	0.43	0.29, 0.63	<0.001
	Above average cigarette smoking^a^		
Number of leisure activity types			
- Active less than weekly or not at all	2.59	1.14, 5.92	0.024
- Active in one activity at least once a week	1		
- Active in two activities at least once a week	0.40	0.25, 0.64	<0.001
- Active in three or four activities at least once a week	0.47	0.26, 0.86	0.015
	Any consumption of other forms of tobacco		
Number of leisure activity types			
- Active less than weekly or not at all	1.53	0.90, 2.61	0.116
- Active in one activity at least once a week	1		
- Active in two activities at least once a week	0.53	0.40, 0.69	<0.001
- Active in three or four activities at least once a week	0.38	0.28, 0.50	<0.001

*OR, odds ratio; CI, confidence interval. All models adjusted for gender, parental education, and parental financial status, family support, availability of friends, and symptoms of mental health problems*.

a *The sample was restricted to respondents that reported smoking any number of cigarettes other than 0 (i.e., sample n = 547)*.

Table 5 displays associations between the number of leisure activity types and alcohol use (general consumption, binge drinking, intoxication, coping with alcohol). Engaging in three or four activities was associated with 46% reduced odds of any alcohol consumption, and among those who consumed alcohol, engaging in three or more activities was associated with 40% reduced odds of above average alcohol consumption. Engaging in three or more activities was associated with 55% reduced odds of engaging in binge drinking, while engaging in three or four activities was associated with 49% reduced odds of above average binge drinking. In terms of the number of occasions being intoxicated, less than weekly activity was associated with 78% increased odds of having been intoxicated. Engaging in three or four activities was associated with 28% reduced odds of having been intoxicated in the first place, while engaging in two or more activities was associated with 48–64% reduced odds of above average number of times having been intoxicated. Finally, engaging in two or more activities was associated with 22–51% reduced odds of having used alcohol as a coping method.

**Table 5:** The association between number of leisure activity types and alcohol use estimated by multivariable logistic regression.

	**OR**	**95% CI**	***P*-value**
	Any alcohol consumption		
Number of leisure activity types			
- Active less than weekly or not at all	1.93	0.89, 4.16	0.093
- Active in one activity at least once a week	1		
- Active in two activities at least once a week	0.80	0.62, 1.03	0.088
- Active in three or four activities at least once a week	0.54	0.39, 0.73	<0.001
	Above average alcohol consumption^a^		
Number of leisure activity types			
- Active less than weekly or not at all	1.63	0.95, 2.79	0.074
- Active in one activity at least once a week	1		
- Active in two activities at least once a week	0.77	0.58, 1.01	0.058
- Active in three or four activities at least once a week	0.60	0.44, 0.82	0.002
	Any binge drinking		
Number of leisure activity types			
- Active less than weekly or not at all	1.31	0.57, 2.98	0.522
- Active in one activity at least once a week	1		
- Active in two activities at least once a week	0.74	0.55, 1.01	0.058
- Active in three or four activities at least once a week	0.45	0.32, 0.64	<0.001
	Above average binge drinking^a^		
Number of leisure activity types			
- Active less than weekly or not at all	1.11	0.61, 1.99	0.736
- Active in one activity at least once a week	1		
- Active in two activities at least once a week	0.84	0.63, 1.12	0.239
- Active in three or four activities at least once a week	0.51	0.36, 0.73	<0.001
	Any occasions being intoxicated^a^		
Number of leisure activity types			
- Active less than weekly or not at all	1.78	1.01, 3.15	0.046
- Active in one activity at least once a week	1		
- Active in two activities at least once a week	0.85	0.67, 1.08	0.175
- Active in three or four activities at least once a week	0.72	0.53, 0.99	0.044
	Above average occasions of being intoxicated^a^		
Number of leisure activity types			
- Active less than weekly or not at all	0.71	0.37, 1.38	0.309
- Active in one activity at least once a week	1		
- Active in two activities at least once a week	0.52	0.36, 0.76	0.001
- Active in three or four activities at least once a week	0.36	0.24, 0.56	<0.001
	Any coping with alcohol^b^		
Number of leisure activity types			
- Active less than weekly or not at all	1.48	0.85, 2.57	0.159
- Active in one activity at least once a week	1		
- Active in two activities at least once a week	0.78	0.61, 0.98	0.036
- Active in three or four activities at least once a week	0.49	0.36, 0.66	<0.001

*OR, odds ratio; CI, confidence interval. All models adjusted for gender, parental education, and parental financial status, family support, availability of friends, and symptoms of mental health problems*.

a *The sample was restricted to respondents that reported consuming alcohol on any number of occasions other than 0 (i.e., sample n = 1,823)*.

b *The sample was restricted to respondents that reported consuming alcohol on any number of occasions other than 0 within the past 12 months (i.e., sample n= 2,180)*.

Table 6 displays the association between number of leisure activity types, drug use and cannabis consumption (any drug use and any cannabis consumption). Engaging in three or four activities was associated with 34% reduced odds of having used drugs. Finally, engaging in two or more activities was associated with 44–47% reduced odds of using cannabis, and among those who consumed cannabis, engaging in three or four activities was associated with 47% reduced odds of above average cannabis consumption. In terms of any drug or cannabis consumption, less than weekly activity was associated with approx. double the odds of the outcomes.

**Table 6:** The association between number of leisure activity types and drug use or cannabis consumption estimated by multivariable logistic regression.

	**OR**	**95% CI**	***P*-value**
	Any drug use		
Number of leisure activity types			
- Active less than weekly or not at all	2.16	1.30, 3.61	0.003
- Active in one activity at least once a week	1		
- Active in two activities at least once a week	0.76	0.54, 1.07	0.120
- Active in three or four activities at least once a week	0.66	0.44, 0.98	0.041
	Any cannabis consumption		
Number of leisure activity types			
- Active less than weekly or not at all	1.95	1.12, 3.39	0.019
- Active in one activity at least once a week	1		
- Active in two activities at least once a week	0.53	0.37, 0.76	0.001
- Active in three or four activities at least once a week	0.56	0.36, 0.87	0.010
	Above average cannabis consumption^b^		
Number of leisure activity types			
- Active less than weekly or not at all	3.20	0.96, 10.6	0.057
- Active in one activity at least once a week	1		
- Active in two activities at least once a week	0.69	0.39, 1.22	0.193
- Active in three or four activities at least once a week	0.53	0.29, 0.95	0.034

*OR, odds ratio; CI, confidence interval. The model adjusted for gender, parental education, and parental financial status, family support, availability of friends, and symptoms of mental health problems*.

b *The sample was restricted to respondents that reported using cannabis on any number of occasions other than 0 (i.e., sample n = 364)*.

Overall, although not all categories reached statistical significance, the pattern of ORs suggest a dose-response relationship, with reduced odds of the outcomes with each increase in the variety of leisure activity types. In a few cases, ORs do not indicate a dose-response relationship (above average cigarette smoking; any cannabis consumption), however, a dose-response relationship for these outcomes could potentially emerge with a larger sample. As a sensitivity analysis (results shown in Appendix 1, Table A1), we performed the same analyses for all outcomes where we entered the predictor variable as a continuous rather than categorical variable. In these models, all results showed significant relationships supporting a dose-response trend between number of leisure activity types and all outcomes.

## Discussion

Our results show that engaging in multiple leisure activity types at least weekly—as compared to a single type of activity weekly—is associated with higher odds for high mental well-being and reduced odds for being very bothered by mental health problems. Being engaged in more leisure activity types is also associated with reduced odds of using alcohol as a coping method, as well as reduced odds for a number of outcomes pertaining to substance use frequency. Specifically, adolescents that engage in multiple leisure activity types are less likely to use substances in the first place, and if they use substances, their use is less likely to be above average. The most robust associations were observed for engaging in three or four activities. Engaging in two activities was not strong enough to reach statistical significance in about half of the models, although they still showed similar associations in terms of ORs. For several outcomes, less than weekly activity was associated with substantial increased odds for numerous substance use outcomes, as compared to one single type of activity weekly. Overall, our results provide an indication of a dose-response relationship, with reduced odds for poor mental health and substance use with increases in the variety of leisure activity types.

### Strengths and Limitations

Some strengths and limitations should be kept in mind before interpreting the results. Major strengths include the use of validated scales for measuring mental well-being and drinking motives, and the use of a large school-based survey. Some limitations are worth mentioning. First, the cross-sectional design precludes us from making inferences regarding directions of causality. Second, this study is based on self-report data, which can lead to recall bias. Third, the school participation rate was low. This was as expected since the schools in Denmark are often overwhelmed by survey requests. Hence, many schools only participate in surveys that are mandatory. In addition, the timing of the survey was not optimal, as it was close to the examination period and many schools thought that the students should focus on their studies at this time of the year. Due to the response rate, we cannot rule out the possibility that some schools characterized by more mental health problems and substance use were not among those participating, and the same may be argued regarding the individual students not participating. We have applied weights in all analyses in order to reduce the bias generated by geographical differences in school participation rates. It may also be noted that the ESPAD target population did not include those who were enrolled in either special schools or special classes for students with learning disorders or severe physical disabilities, as described in the sampling guidelines for the ESPAD project (www.espad.org). Fourth, actual parental income was not available in our dataset, and we could therefore not adjust for it. The variable used for parental income pertains to participants' perceived income status relative to peers, which arguably presents some limitations as compared to an actual income measure. It may also be noted that the variable for parental education was characterized by 23.4% of missing data. We created a “missing” category in order to be able to include participants in these models even if data for this variable was missing. It is possible that the limitations of these two variables could have influenced the results, since higher education and wealth may result in both increased access to activities and better mental health for adolescents. Finally, we did not have access to validated scales for specific mental health problems (e.g., depression, anxiety), and therefore used items pertaining to experienced symptoms.

### Contextualization of Findings

In line with previous studies, our results suggest that adolescents that engage in multiple leisure activity types may be protected against engaging in substance use. These activities may reduce available time that otherwise could be spent on using substances (Murphy et al., 2006; Audrain-McGovern et al., 2013; Leventhal et al., 2015). Importantly, our results suggest that not only are more active adolescents less likely to use substances in the first place, but among adolescents that already use substances, those that are more active are less likely to engage in above-average substance use. A recent systematic review of 50 research studies confirms an inverse relationship between engaging in substance-free activities and substance use (Acuff et al., 2019). Further, the protective effects of being engaged in activities is also suggested in Iceland, where systematic efforts to increase afterschool and evening alternatives for teens has resulted in substantial reductions in adolescent substance use and misuse (Kristjansson et al., 2010, 2016).

It is not just the elimination or reduction in opportunities to engage in substance use that has a role to play, but the literature also emphasizes the role of alternative reinforcing leisure activities. Our results support the notion of leisure activity operating as an alternative reinforcer since engaging in multiple leisure activity types was positively related to mental well-being, inversely related to mental health problems, and inversely related to using substances as a coping method. This means that what these leisure activities may offer, such as promoting new skills, self-esteem, meaning, and purpose, or opportunities to form interpersonal relationships, may enhance mental health and well-being, which in turn is likely to reinforce leisure activity (Kleiber and Kirshnit, 2002; Santini et al., 2020). Leisure activities may also serve as a constructive way to cope with the stress and difficulties often experienced by adolescents (Kleiber, 1999; Iwasaki and Mannell, 2000; Trenberth and Dewe, 2002). If adolescents use leisure activities as a healthy coping strategy, it may reduce an otherwise existing need to use substances to cope with difficulties.

Not all adolescents have access to a variety of appealing leisure activities (e.g., due to socioeconomic disadvantage or for example living in rural areas with limited options), or they are not being motivated to engage in leisure activities through their upbringing or their social or educational context (Leventhal et al., 2015; Andrabi et al., 2017). Previous research on substance use among adolescents highlights the importance of deficits in substance-free reinforcement in the development and maintenance of drug addiction in addition to the reinforcing efficacy of the drug itself (i.e., the lack of alternative rewarding substance-free activities exacerbates the reinforcing properties of a drug) (Acuff et al., 2019). Other research has shown that diminished access to and engagement in rewarding substance-free activity may lead to substance use and misuse in adolescence (Leventhal et al., 2015; Andrabi et al., 2017). In other words, increasing opportunities to engage in substance-free enjoyable activities is suggested to be useful in substance use prevention among adolescents.

Since our cross-sectional design precludes us from making inferences regarding the direction of associations, it is relevant to consider the possibility that our results may suggest the opposite direction of associations, namely that mental health problems and substance use predict less engagement in leisure activities. This scenario is also problematic, since the lack of engagement in leisure activities may (1) in and of itself be a sign of unhealthy or inhibited bio-psycho-social development in adolescence, and (2) translate into inactive lifestyles, social withdrawal and isolation, or the replacement of leisure activities with risk behaviors or delinquency (Wegner and Flisher, 2009; Caldwell and Faulk, 2013; Spaeth et al., 2015). Future longitudinal and intervention research is needed to assess the extent to which multiple leisure activity types may be protective against mental health problems and substance use, in particular research designs that make it possible to assess uni-directional or bi-directional influences between leisure activities and mental health/substance use.

### Implications for Policy and Practice

Leisure activity is often thought of as being a matter of pleasurable recreation, but often—perhaps with the exception of sports—not as something that can protect and promote human health (Donovan et al., 2007; Wilcox et al., 2009). This study builds on previous health promotion research showing that there is a pertinent need to promote such activities as being health promoting, and particularly so during developmental stages where core values and habits are formed (Donovan and Anwar McHenry, 2014; Koushede et al., 2015; Santini et al., 2018b). It is critical to prevent adverse health outcomes in the adolescent population by the promotion of behaviors known to promote mental health through (1) active lifestyles, (2) social connectedness, and (3) meaningful commitment or contribution to society. While there is a multitude of behavioral factors that may benefit mental health, a number of studies have shown that these three behavioral domains—known as Act-Belong-Commit—promote positive mental health in adulthood as well as protect against mental, neurological, and alcohol use disorders (Nielsen et al., 2017; Santini et al., 2017a, 2018a). Act-Belong-Commit, also referred to as *the ABCs of Mental Health*, is a mental health campaign and framework for doing mental health promotion, which is currently being deployed in different parts of the world (Koushede et al., 2015; Santini et al., 2018). Importantly, it has shown promising results in school settings in Australia, where is has been fruitful in promoting mental health and well-being among both students as well as teachers (Anwar-McHenry et al., 2016; Anwar McHenry et al., 2018). Our study provides further evidence suggesting that similar policy and practice efforts may do well to promote and increase access to leisure activities among adolescents.

## Conclusion

Among adolescents in Denmark, our results show that engaging in multiple leisure activity types at least weekly—as compared to one single type of activity—is associated with increased odds of high mental well-being and reduced odds of being very bothered by mental health problems. Further, adolescents that engage in multiple leisure activity types are less likely to use substances in the first place, less likely to use substances to cope, and if they use substances, their consumption is less likely to be above average. Increasing opportunities for adolescents to engage in rewarding substance-free activities is suggested to be useful in mental health promotion and the prevention of excessive use of substances. Policy and practice efforts may promote and increase access to leisure activities among adolescents.

## Data Availability Statement

We do not have permission to share data. Access must be obtained via the ESPAD website. Requests to access these datasets should be directed to http://www.espad.org/organisation-contacts.

## Ethics Statement

This study is a secondary data analysis with no human subject issues. Written informed consent from the participants' legal guardian/next of kin was not required to participate in this study in accordance with the national legislation and the institutional requirements.

## Author Contributions

All authors have contributed to the work and approved it for publication.

## Conflict of Interest

The authors declare that the research was conducted in the absence of any commercial or financial relationships that could be construed as a potential conflict of interest.
