# Supplementary material for: Associations Between Multiple Leisure Activities, Mental Health and Substance Use Among Adolescents in Denmark: A Nationwide Cross-Sectional Study
Source: Front Behav Neurosci. 2020 Dec 21;14:593340. doi: 10.3389/fnbeh.2020.593340 (PMC7779398; doi:10.3389/fnbeh.2020.593340)
Supplement: Supplementary file 1 [file Data_Sheet_1.pdf]

Appendix 1

List of outcomes

Mental health outcomes: symptoms of mental health problems were assessed by asking respondents which of the following symptoms they had been bothered by within the past two weeks: 1) fatigue; 2) sleep problems or insomnia; 3) melancholy, depression or unhappiness; 4) anxiety, nervousness, restlessness or apprehension (1). Response options were: 0 not bothered; 1 yes, bothered to some extent; 2 yes, very bothered. For the continuous measure (to be used as a covariate), responses were summed up to a continuous scale ranging from 0 (none) to 8 (very bothered by all). For the binary measure (to be used as an outcome), mental health problems (very bothered) was operationalized as present if the participant had answered yes to having been very bothered by any of the four symptoms, and absent if not.

The Short Warwick-Edinburgh Mental Well-being Scale (SWEMWBS) is a well validated measure used to monitor mental well-being amongst a population is based on a conceptualisation of mental well-being as feeling good and functioning well. The scale has recently been validated in Denmark (2). SWEMWBS consists of 7 positively worded questions: 1) I've been feeling optimistic about the future, 2) I've been feeling useful, 3) I've been feeling relaxed, 4) I've been dealing with problems well, 5) I've been thinking clearly, 6) I've been feeling close to other people, 7) I've been able to make up my own mind about things. Response options were: none of the time; rarely; some of the time; often; all of the time. Summing up the scale leads to a score between 7 and 35; the higher the score, the higher mental well-being. We used a similar approach as in previous studies (3, 4), where high mental well-being was defined as the top 15th percentile with a SWEMWBS score ≥27.03.

Smoking:

In terms of cigarettes, participants were asked "How often have you smoked cigarettes (excluding e-cigarettes) during the last 30 days?", with the following response options: not at all; less than 1 cigarette per week; less than 1 cigarette per day; 1-5 cigarettes per day; 6-10 cigarettes per day; 11-20 cigarettes per day; more than 20 cigarettes per day. Any cigarette smoking was coded as present if the participant had answered anything other than "not at all". Above average cigarette smoking was coded as present when the participant reported smoking 1 cigarette per day or more (applying to 43.4% of participants).

Participants were also asked "Have you ever used e-cigarettes", "Have you ever used waterpipe?", and "Have you ever used heat-not-burn tobacco?" Consuming any of these three possibilities within the last 12 months was coded a present, and absent if not.

Finally, participants were asked "On how many occasions (if any) have you used cannabis during the last 12 months?", with the following response options: 0; 1-2; 3-5; 6-9; 10-19; 20-39; 40 or more. Any cannabis consumption was coded as present if the participant had answered anything other than "0". Above average cannabis consumption was coded as present when the participant reported having used cannabis on 6 occasions or more (applying to 44.2% of participants).

Alcohol consumption:

In terms of general alcohol consumption, participants were asked “On how many occasions (if any) have had any alcoholic beverage to drink during the last 30 days?”, with the following response options: 0; 1-2; 3-5; 6-9; 10-19; 20-39; 40 or more. Any alcohol consumption was coded as present if the participant had answered anything other than “0”. Above average alcohol consumption was coded as present when the participant reported having consumed alcohol on 6 occasions or more (applying to 37.5% of participants).

In terms of binge drinking, participants were asked “Think back again over the last 30 days. How many times (if any) have had five or more drinks on one occasion?” “A `drink´ is defined as 1 bottle/can of beer, 1 bottle/can of cider, 1 bottle of premixed drinks, 1 glass of wine, 1 glass of spirits (ca 4 cl)”. Response options were: None; 1; 2; 3-5; 6-9; 10 or more times. Any binge drinking was coded as present if the participant had answered anything other than “0”. Above average binge drinking was coded as present when the participant reported having engaged in binge drinking on 3 occasions or more (applying to 46.8% of participants).

In terms of occasions of being intoxicated, participants were asked “On how many occasions during the last 30 days (if any) have you been intoxicated from drinking alcoholic beverages, for example staggered when walking, not being able to speak properly, throwing up or not remembering what happened?”, with the following response options: 0; 1-2; 3-5; 6-9; 10-19; 20-39; 40 or more. Any occasions of being intoxicated was coded as present if the participant had answered anything other than “0”. Above average occasions of intoxication was coded as present when the participant reported having consumed alcohol on 3 occasions or more (applying to 32.1% of participants).

Finally, in terms of using alcohol as a coping method, three items from the Short-Form Drinking Motives Questionnaire (5) was used. Participants were asked how often in the last 12 months they drank: 1) “because it helps you when you feel depressed or nervous?”, 2) “to cheer up when you're in a bad mood?”, 3) “to forget about your problems?” Responses options were: never; seldom; sometimes; mostly; always. Using alcohol as a coping method was coded as present if the participant responded at least “seldom” on any of the three items, and absent if not.

Drug consumption:

In terms of drug consumption, participants were asked on how many occasions in their lifetime (if any) they had used: 1) ecstasy, 2) amphetamines, 3) methamphetamines, 4) cocaine, 5) crack, 6) heroin, 7) inhalants, 8) hallucinogens, 9) magic mushrooms, 10) gamma-hydroxybutyrate (GHB), 11) injection, 12) tranquilizers, 13) pills with alcohol to get high, 14) painkillers to get high, or 15) nitrous oxide cartridges. Response options were: 0; 1-2; 3 or more. Any drug consumption was coded as present if the participant had answered anything other than “0” to any of the 15 items.

Missing data:

All statistical models were based on the sample with no missing data (complete case analysis), and the proportion of missing data within the study sample (N=2,488) were as follows: number of leisure activities 1.8%; gender 0.0%; parental education 0.0% (see methods section); family support 2.2%; availability of friends 2.5%; parental income status 2.5%; mental health problems 7.3%; mental well-being 7.4%; cigarette smoking 0.0%;

waterpipe and heat-not-burn tobacco consumption 0.1%; cannabis consumption 0.2%; general alcohol consumption 1.1%; binge drinking 0.2%; intoxication 1.2%; using alcohol as a coping method 0.9%; drug consumption 1.6%.

Table A1. The association between number of leisure activity types (continuous) and all outcomes estimated by multivariable logistic regression.

	**OR**	**95%CI**	**P-value**
	Mental health problems (very bothered)[a]		
Number of weekly leisure activities (range 0-3)	0.85	0.76, 0.96	0.008
	High mental well-being[b]		
Number of weekly leisure activities (range 0-3)	1.30	1.12, 1.51	0.001
	Any cigarette smoking		
Number of weekly leisure activities (range 0-3)	0.62	0.52, .74	< 0.001
	Above average cigarette smoking[a]		
Number of weekly leisure activities (range 0-3)	0.58	0.45, .75	< 0.001
	Any consumption of other forms of tobacco		
Number of weekly leisure activities (range 0-3)	0.61	0.53, 0.70	< 0.001
	Any alcohol consumption		
Number of weekly leisure activities (range 0-3)	0.71	0.61, 0.83	< 0.001
	Above average alcohol consumption[b]		
Number of weekly leisure activities (range 0-3)	0.75	0.65, 0.87	< 0.001
	Any binge drinking		
Number of weekly leisure activities (range 0-3)	0.68	0.57, 0.80	< 0.001
	Above average binge drinking[b]		
Number of weekly leisure activities (range 0-3)	0.75	0.64, 0.87	< 0.001
	Any occasions being intoxicated[a]		
Number of weekly leisure activities (range 0-3)	0.81	0.70, 0.94	0.006
	Above average occasions of being intoxicated[a]		
Number of weekly leisure activities (range 0-3)	0.68	0.57, 0.81	< 0.001
	Any coping with alcohol[c]		
Number of weekly leisure activities (range 0-3)	0.70	0.61, 0.79	< 0.001
	Any drug use		
Number of weekly leisure activities (range 0-3)	0.75	0.62, 0.90	0.002
	Any cannabis consumption		
Number of weekly leisure activities (range 0-3)	0.70	0.57, 0.87	0.002
	Above average cannabis consumption[d]		
Number of weekly leisure activities (range 0-3)	0.65	0.50, .85	0.002

The model with mental health problems as outcome adjusted for gender, parental education, and parental financial status, family support, and availability of friends. All other models included the same covariates as well as symptoms of mental health problems.
[a] The sample was restricted to respondents that reported smoking any number of cigarettes other than 0 (i.e. sample n=547).
[b] The sample was restricted to respondents that reported consuming alcohol on any number of occasions other than 0 (i.e. sample n=1,823).
[c] The sample was restricted to respondents that reported consuming alcohol on any number of occasions other than 0 within the past 12 months (i.e. sample n=2,180).
[d] The sample was restricted to respondents that reported using cannabis on any number of occasions other than 0 (i.e. sample n=364).

References:

1. Jensen HAR, Davidsen M, Ekholm O, Christensen AI. Danskernes sundhed: Den Nationale Sundhedsprofil 2017. København: Sundhedsstyrelsen; 2018.
2. Koushede V, Lasgaard M, Hinrichsen C, Meilstrup C, Nielsen L, Rayce SB, et al. Measuring mental well-being in Denmark: validation of the original and short version of Warwick-Edinburgh Mental Well-Being Scale (WEMWBS and SWEMWBS) and cross-cultural comparison across four European settings. Psychiatry research. 2019;271:502-9.
3. Stewart-Brown S, Samaraweera PC, Taggart F, Kandala N, Stranges S. Socioeconomic gradients and mental health: implications for public health. The British Journal of Psychiatry. 2015;206(6):461-5.
4. Santini ZI, Stougaard S, Koyanagi A, Ersbøll AK, Nielsen L, Hinrichsen C, et al. Predictors of high and low mental well-being and common mental disorders: findings from a Danish population-based study. European journal of public health. 2020.
5. Harbke CR, Laurent J, Catanzaro SJ. Comparison of the Original and Short Form Drinking Motives Questionnaire–Revised With High School and Underage College Student Drinkers. Assessment. 2019;26(7):1179-93.
